# Supplementary material for: Prevalence of and Impact on the Outcome of Myosteatosis in Patients with Hepatocellular Carcinoma: A Systematic Review and Meta-Analysis
Source: Cancers (Basel). 2024 Feb 27;16(5):952. doi: 10.3390/cancers16050952 (PMC10930530; doi:10.3390/cancers16050952)
Supplement: Supplementary file 1 [file cancers-16-00952-s001.zip › Supplementary Figures.pdf]

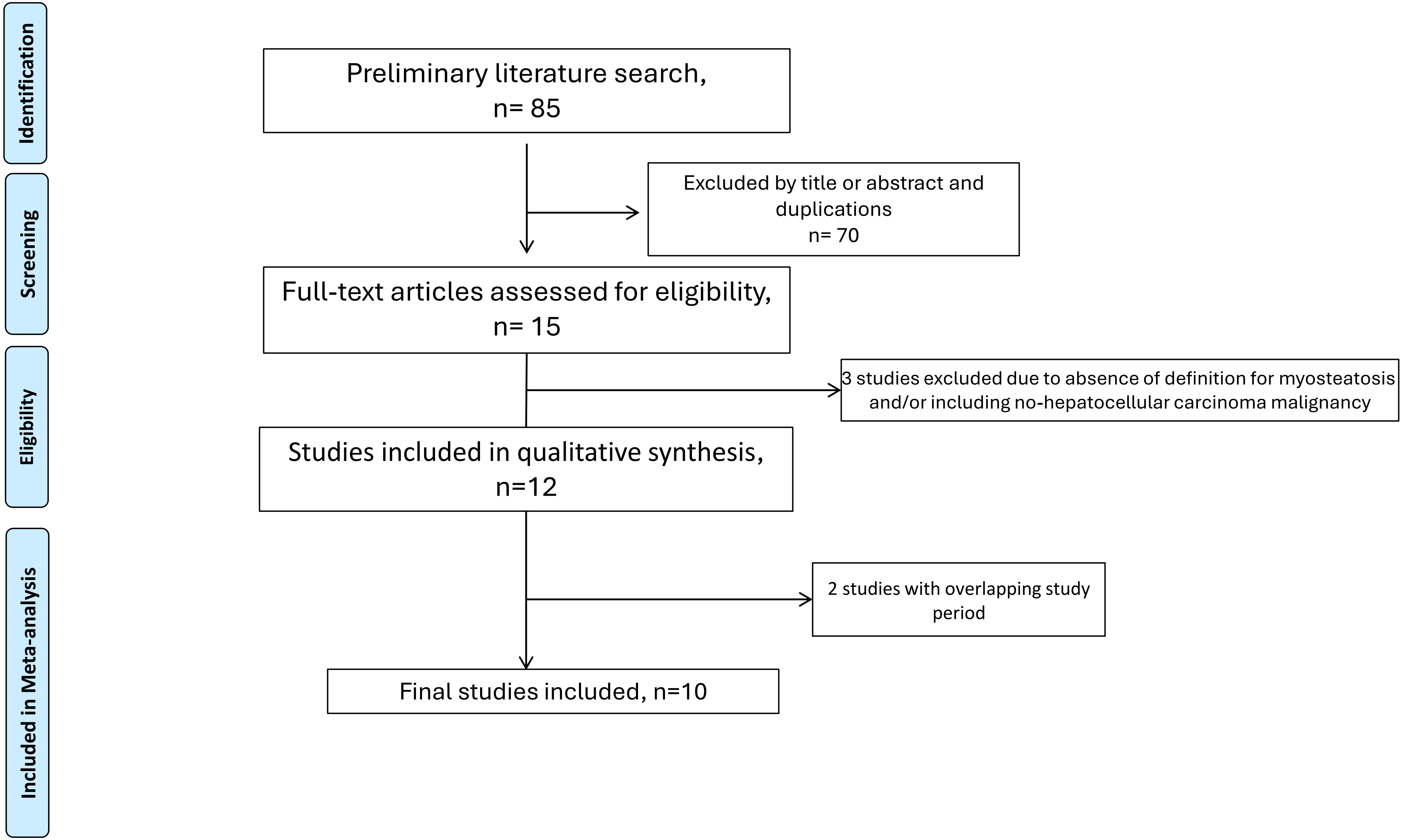

Suppl. Figure S1. PRISMA Flow diagram of study selection

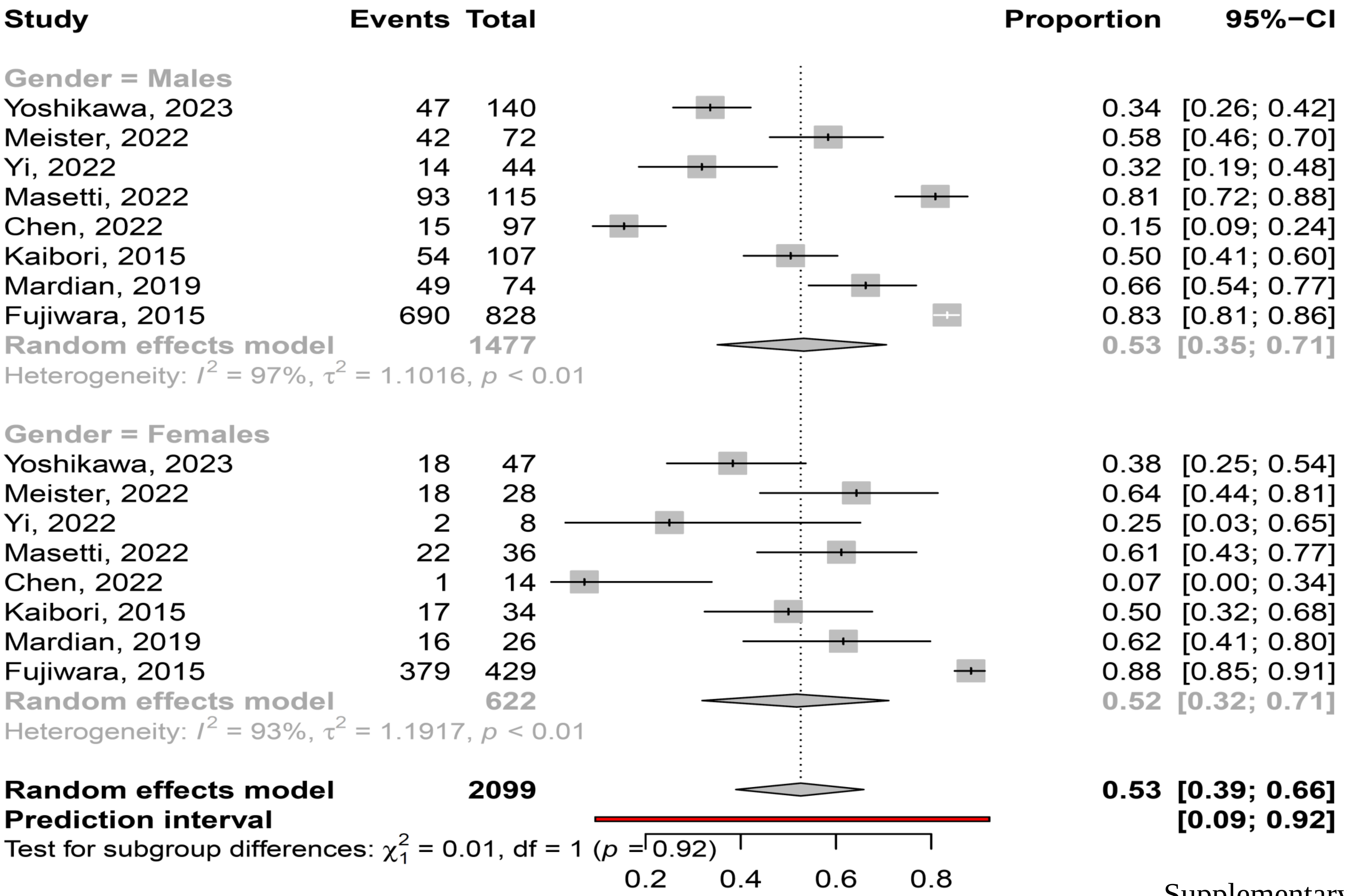

Supplementary FIGURE S2

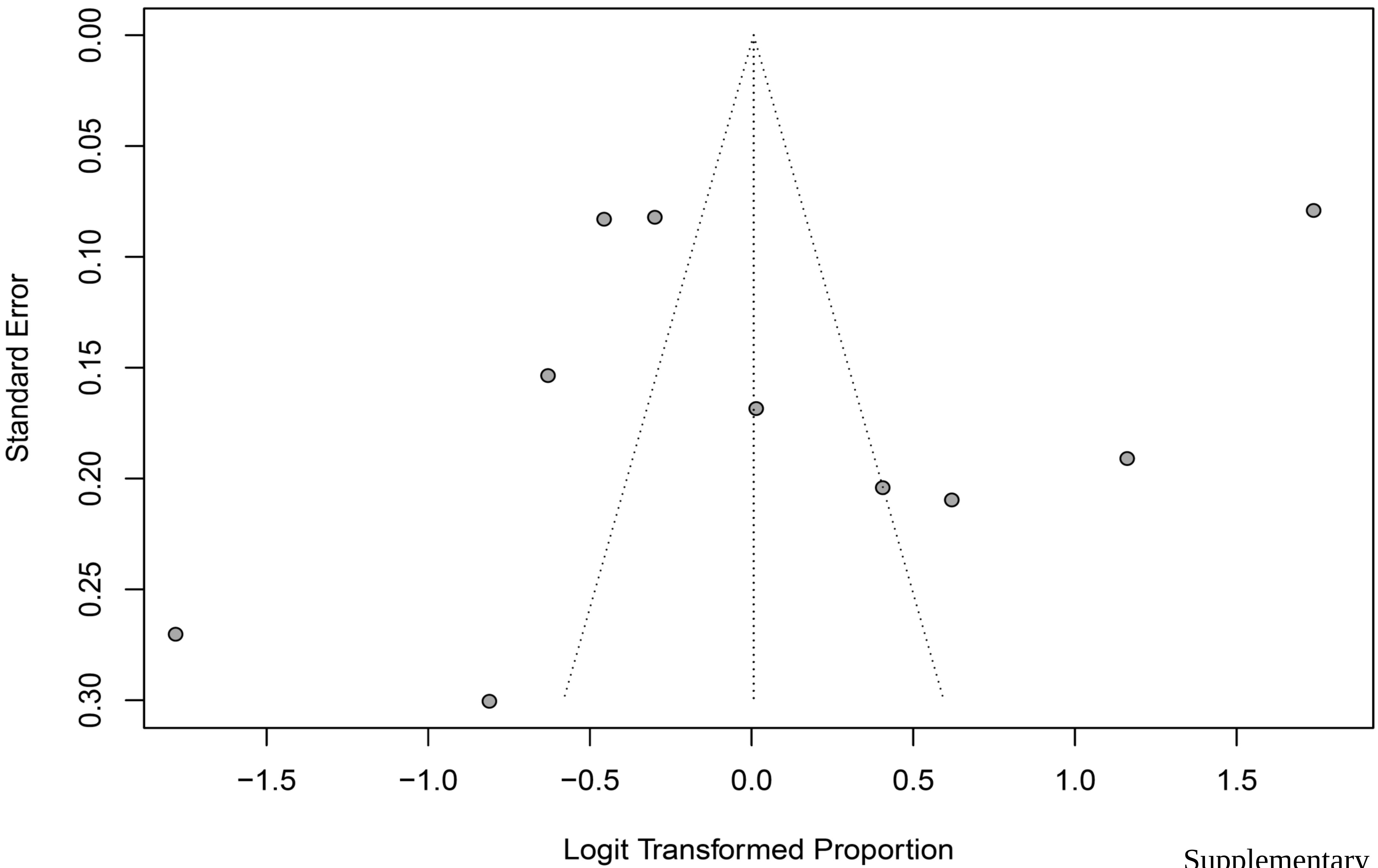

Supplementary FIGURE S3
