# Supplementary material for: Prevalence of and Impact on the Outcome of Myosteatosis in Patients with Hepatocellular Carcinoma: A Systematic Review and Meta-Analysis
Source: Cancers (Basel). 2024 Feb 27;16(5):952. doi: 10.3390/cancers16050952 (PMC10930530; doi:10.3390/cancers16050952)
Supplement: Supplementary file 1 [file cancers-16-00952-s001.zip › Supplementary Table S1 MYOSTEATOSIS HCC.pdf]

Table S1. Published studies regarding the characteristics of HCC patients with myosteatosis, compared to those without myosteatosis.

First author, Country, Publication year, Study design (Ref.)	Newcastle-Ottawa Scale*	Definition of myosteatosis	Number of patients n/n	Male sex, n/n	Alcohol n/n	NAFLD n/n	Viral hepatitis n/n	Diabetes mellitus, n/n	CP class A, B, C n/n	Complications, n/n	Recurrence/no response, HCC n/n	Death, n/n	Follow up, months
Yoshikawa, Japan, 2023, RS [11]	8 (4/1/3)	Gender-based	65/122	47/93	NA/NA	NA/NA	32/70	24/78	63,2,0/120, 2, 0	0/1	18/34	NA/NA	60
Bannangkoon, Thailand, 2023, RS [12]	7 (3/1/3)	Gender-based	237/374	NA/NA	NA/NA	NA/NA	NA/NA	NA/NA	NA/NA	51/72	104/117	215/287	48
Meister, Germany, 2022, RS [13]	8 (4/1/3)	BMI-based	60/40	42/30	23/5	24/14	8/17	NA/NA	NA/NA	15/2	NA/NA	NA/NA	52
Yi, China, 2022, RS [14]	6 (3/1/2)	Gender-based	16/36	14/30	NA/NA	NA/NA	14/28	NA/NA	14,2,0/28, 8, 0	NA/NA	NA/NA	NA/NA	10
Fujiwara, Japan, 2015, PS [15]	9 (4/2/3)	Gender-based	1069/188	690/138	164/27	NA/NA	881/169	270/40	820,237,12/138,49,1	NA/NA	NA/NA	774/97	60
Masetti, Italy, 2022, RS [16]	8 (4/1/3)	Gender-based	115/36	93/22	30/4	19/4	58/18	34/11	99, 15, 1/ 25, 11, 0	9/2	NA/NA	65/20	NA
Hamaguchi, Japan, 2019, RS [17]	8 (4/1/3)	Gender-based	258/348	NA/NA	NA/NA	NA/NA	NA/NA	NA/NA	NA/NA	NA/NA	179/278	NA/NA	NA
Chen, Taiwan, 2022, RS [18]	7 (3/1/3)	BMI-based	16/95	15/82	NA/NA	NA/NA	12/89	NA/NA	16, 0, 0/16, NA, NA	NA/NA	11/28	11/33	10
Kaibori, Japan, 2015, RS [19]	7 (3/1/3)	Gender-based	71/70	54/53	NA/NA	NA/NA	54/54	21/10	65, 6, 0/70, 0, 0	11/4	61/54	41/34	48
Mardian, Indonesia, 2019, PS [20]	8 (4/1/3)	Gender-based	65/35	49/25	NA/NA	18/16	47/19	NA/NA	29, 29, 7/31, 3, 1	NA/NA	NA/NA	60/17	NA

HCC; hepatocellular carcinoma; NA not available; RS: retrospective study, PS: prospective study; BMI: body mass index; NAFLD: non alcoholic fatty liver disease; CP: Child-Pugh; mo: months.

All n/n results: number of patients with myosteatosis / number of patients without myosteatosis

*in parenthesis for Newcastle-Ottawa Scale (selection [max 4]/comparability [max 2]/outcome [max 3]).
